# Functional mesoporous materials in clean energy: an interview with Dongyuan Zhao

**DOI:** 10.1093/nsr/nwad062

**Published:** 2023-03-06

**Authors:** He Zhu

**Affiliations:** NSR, Beijing, China

## Abstract

*National Science Review* invited Prof. Dongyuan Zhao of Fudan University for an interview focusing on his team's renowned research on functional mesoporous materials and energy-related applications. Prof. Zhao is a professor of chemistry and materials science, and a member of the Chinese Academy of Sciences. He received his PhD in chemistry from Jilin University in 1990. He has since focused his research on the synthesis and structure of porous materials and molecular sieves. His team received a first-tier national science award in 2021 for their contribution to the research and development of mesoscopic materials. They discovered a method of synthesizing mesoporous organic polymers and carbonaceous materials using organic-organic self-assembly. This work was published in 2005 and since then it has turned into a vibrant new field of more than 40 000 publications so far. His team has named more than 20 of their inventions after Fudan University: the FDU mesoporous series.

## THE SYNTHESIS OF MESOPOROUS CARBON

***NSR**:* Prof. Zhao, thank you for making time for this interview. Would you like to briefly describe what mesoporous materials are?

*Zhao:* When we talk about mesoporous materials, we should first mention nanomaterials. Their development started at the beginning of the 21st century. They possess surface effects, microscopic properties and macroscopic quantum tunneling properties. Porous materials are matrix or framework structures with connected or isolated voids or pores that can be filled with liquid or gas. Porous materials often have large surface areas and interconnected paths for the transport and diffusion of materials. According to the definition of the International Union of Pure and Applied Chemistry (IUPAC), mesoporous materials contain pores between 2 nm and 50 nm in size. Below 2 nm, it is called a micropore; above 50 nm, it is called a macropore. Pore structures in the *meso* range produce extraordinary effects. For example, pore walls less than 20 nm and channels shorter than 100 nm may significantly shorten the transport distance of electrons and ions. This may greatly enhance the efficiency of water-splitting reactions and energy-storage devices such as batteries and supracapacitors.

I saw the similarity between my work [in graduate school] and this discovery and realized I had come so close to a breakthrough like that.

—Dongyuan Zhao

**Figure 1:**
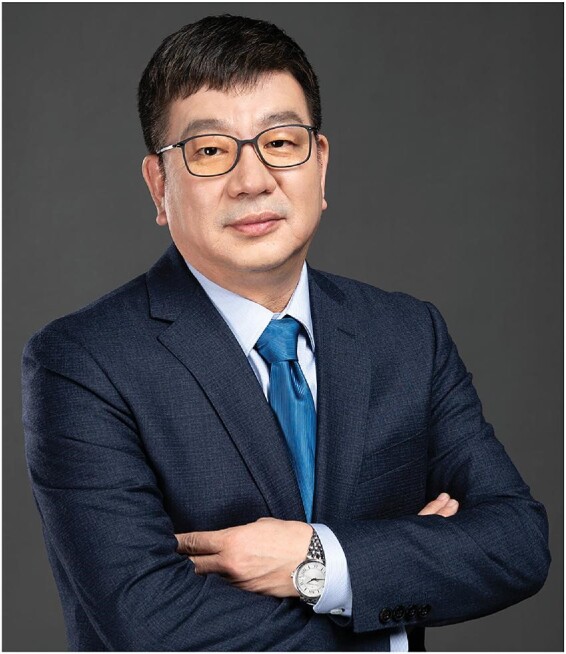
Dongyuan Zhao and his team received a first-tier national science award in 2021 for their contribution to the research and development of mesoscopic materials (*courtesy of Prof. Dongyuan Zhao*).

***NSR**:* How did you get started on this topic?

*Zhao:* My major in graduate school at Jilin University was physical chemistry. My PhD thesis was on the synthesis of pillared-layer molecular sieves. In 1992, the petroleum company Mobil discovered ordered mesoporous materials and published this in *Nature* and *Journal of the American Chemical Society* (JACS). I saw the similarity between my work and this discovery and realized I had come so close to a breakthrough like that. Inspired by Mobil's discovery, I set my goal for that moment as a breakthrough publication in JACS. I started my research in mesoporous materials in 1993 in Israel and published my first article on this topic in 1995. I reached and surpassed that goal a few years after that. As of now I have been working on this for about 30 years.

**Figure 2:**
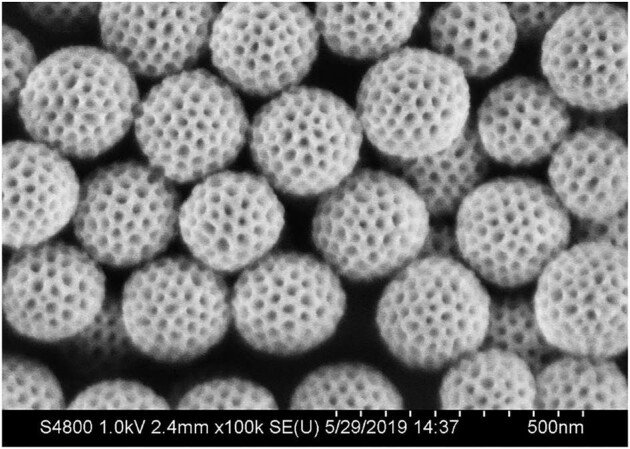
An image of mesoporous carbon acquired by Scanning Electron Microscopy (SEM) (*credit: Zhao Research Group*).

***NSR**:* Would you like to briefly describe the method of synthesizing organic mesoporous materials?

*Zhao:* We use a method called soft-templating. With this method, a constructed material precursor is first mixed with amphiphilic surfactant templates in solution. When the surfactant template is removed, what remains are the porous materials. The critical step is the interaction between the organic monomer and the surfactant molecules. By carefully controlling this step, various sizes and shapes of pores can be obtained. Initially we encountered some difficulty in choosing the right molecular template for polymerization because there were so many organic options. Later we realized the focus should be materials with covalent bonds such as phenol/formaldehyde resin. With that, the process can be started simply by heating.

***NSR**:* What are some of the energy-related applications?

*Zhao:* One major application is using mesoporous materials as a catalyst carrier in petroleum refinery. These materials have advantageous properties such as large and uniform pores and large surface areas. So they can be used as molecular sieves for crude oils with particularly large molecules. We are currently collaborating with SinoPEC to commercialize these materials for the refinery industry. Another important application is in the manufacturing of battery cathodes. The conventional material for that is graphite, which has relatively slower electron transport due to the lack of pores. Now my team is developing new cathode materials using mesoporous carbon-silica. The process of industrializing this technology is well underway, with a location finalized. We are aiming to build the most advanced platform of cathode materials. Other applications include capacitors and catalysts for water-splitting reactions.

If we are able to reach carbon neutrality for the whole world and we can implement large-scale carbon capture to reduce the excessive CO_2_ that is already in the atmosphere, we may be able to make a positive impact on climate change.

—Dongyuan Zhao

## MESOPOROUS MATERIALS IN CARBON CAPTURE

***NSR**:* Now let's focus on the energy sector and start with carbon capture. What is carbon capture and why is it so important with regard to clean energy and climate change?

*Zhao:* We all know China has set the following goals: peak carbon emissions in 2030 and carbon neutrality in 2060. These time points were chosen because in the foreseeable future we have to rely on fossil fuels as our primary energy source. Fossil fuels include natural gas, oil and coal. Based on them, we have built a robust and economic infrastructure to generate electricity and to power transportation. The price we pay is CO_2_ emission. Burning 1 ton of coal generates 3.7 tons of CO_2_. This ever-growing amount of CO_2_ in the atmosphere causes the greenhouse effect and heats up the planet. In addition, from a chemist's point of view, carbon is the foundation of life. All carbon on Earth needs to recycle. If carbon only goes one way, from underground to the atmosphere, eventually we will run out of life.

Now, how do we capture CO_2_ in the air and put it back in the ground? This is a difficult problem not in terms of scientific principles but in terms of the lack of practical methods to scale it. One proposal is to absorb CO_2_ and then compress it and inject it into the ground. Mesoporous materials may be a great absorbent in this process as their high surface area may enable pure CO_2_ to be stored at high density. This is a physical process as there is no chemical reaction needed between CO_2_ and something else. So it may be implemented with high efficiency.

**Figure 3:**
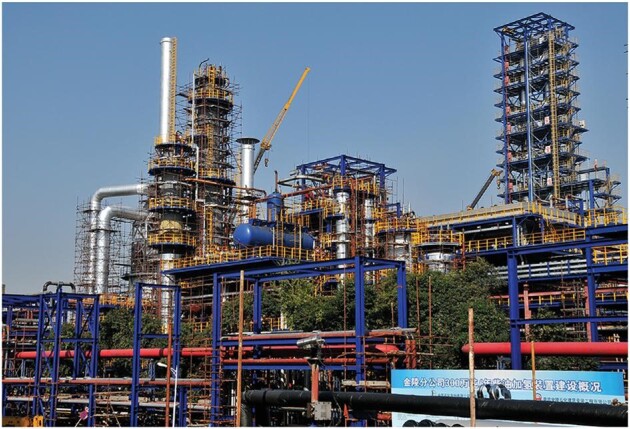
A petroleum refinery of SinoPEC that uses technology developed by Prof. Dongyuan Zhao (*credit: Zhao Research Group*).

For example, mesoporous molecular sieves and metal-organic frameworks (MOFs) could be used for this purpose.

***NSR**:* For carbon capture, what is the difference between using organic and inorganic mesoporous materials?

*Zhao:* The main difference is that organic materials are more absorbent of CO_2_ as they are more similar. Currently, the best choices are MOFs and carbon nanotubes.

## MESOPOROUS MATERIALS IN ENERGY GENERATION AND STORAGE

***NSR**:* Batteries are another hot topic as electric cars are gaining in popularity worldwide. Electric car owners often complain about the low capacity and short lifetime of their batteries. Why is battery technology still so poor after so many years of development?

*Zhao:* First of all, the batteries we have today are orders of magnitude better than before. But people's expectations are also much higher. For example, early electric cars were luxury items for leisure so they did not have to have a range comparable to petrol cars. Now they are meant to be family cars for everyday use. For that purpose, we still feel that the charging time is too long, range is not adequate and short lifetime leads to a high cost of ownership. To overcome these issues, I think first we need to rethink the principles of electrochemistry. Despite the progress we have made, the basic structure of a battery has not changed much since its invention more than 250 years ago. To meet today's demands, we need to design new battery structures with new electrochemical processes. Without fundamental change, it would be difficult for electric cars to replace petrol cars. Second, we need to focus on the problem of internal degradation to address the lifetime problem. We are exploring a lot of new materials, particularly for cathodes. Our target is at least 1000 charge cycles with a range between 500 and 800 kilometers.

I am involved in the review process for a lot of battery-related manuscripts for *National Science Review*. I often recommend that authors emphasize innovative principles. If a manuscript presents a new principle, even if it is not practical, it may inspire new ideas for other people. I would consider it a good paper. On the other hand, if one particular material leads to better experimental results without explanation, I do not see a great value in publishing it. In order to achieve the breakthrough we need in battery technology, we may need the efforts of an entire generation of researchers.

***NSR**:* That is an important insight. Next we turn to solar energy. First of all, how does light turn into electricity? What is the role of porous materials in this process?

*Zhao:* Light can be considered as electromagnetic waves or particles. As particles or photons, its energy can be absorbed by the electrons in a semiconductor and move in the lattice to create electron-hole pairs. As electrons and holes are transported and accumulate, they generate a voltage and a current if a circuit is closed. Mesoporous materials are excellent photocatalysts because they provide a larger surface exposure to light so the photovoltaic process happens with a higher efficiency. A well-known problem with the photovoltaic process is that it is intermittent so it is not a reliable power source. To solve this problem, we can add water electrolysis after the photovoltaic process. As electrical energy is transferred into hydrogen in gas or liquid form, it can be stored and transported more conveniently. In the water-splitting process, mesoporous semiconductors show a higher solar-to-hydrogen efficiency because they have different electron band properties.

As for scientists, if we can revolutionize battery technology, if we can popularize ‘liquid sunshine’, if we can crack artificial photosynthesis, we will change the world.

—Dongyuan Zhao

***NSR**:* Yes. As you explained earlier, the battery technology is still not a great option so we consider using hydrogen as storage. How is this solution actually implemented in practice?

*Zhao:* The photovoltaic industry has received a lot of investment in recent years. In terms of turning it into ‘liquid sunshine’, Prof. Can Li at the Dalian Institute of Chemical Physics currently leads an enterprise in Ningxia that is building a complete supply chain to commercialize photovoltaic hydrogen. On the other hand, the global supply chain of petroleum we rely on today has been built in the course of more than a century. That includes oil fields, refineries and gas stations. Compared to that, the infrastructure of clean energy or renewable energy is still in an early stage. We are far from a turning point where we can transition off fossil fuels. The only way to reach that point, in my opinion, is to organize a concerted, systematic effort on a global scale. We can build a smaller model as a proof-of-concept but to make it commercially viable, we all have to adapt to it.

**Figure 4:**
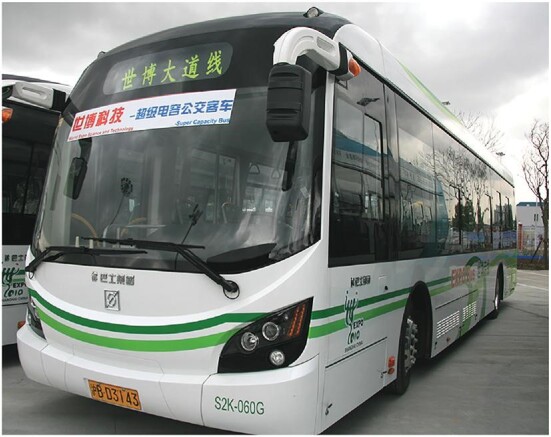
A Shanghai city bus powered by super capacitors (*credit: Zhao Research Group*).

As I mentioned, carbon neutrality and carbon capture are two major components. If the whole world is able to reach carbon neutrality and we can implement large-scale carbon capture to reduce the excessive CO_2_ that is already in the atmosphere, we may be able to make a positive impact on climate change. As for scientists, if we can revolutionize battery technology, if we can popularize ‘liquid sunshine’, if we can crack artificial photosynthesis, we will change the world. So I am a big proponent of basic science research in electrochemical and photochemical processes.

***NSR**:* The next topic is super capacitors. What are they and how are they different from batteries?

*Zhao:* Electricity is stored in a super capacitor as accumulated charges, without any chemical reaction. In a battery, a chemical reaction occurs when ions receive electrons and form atoms. In a super capacitor, electrons simply move and accumulate. The upside is charging and discharging are fast; the downside is that its capacity is much lower than that of a battery. The advantage of mesoporous materials in this application is again that the large surface area allows more charges to be accumulated. In an electric vehicle, a super capacitor can operate in conjunction with a battery to conserve power and increase performance. During braking, the kinetic energy can be turned into electricity and stored in a super capacitor temporarily. This extra power can be released to compensate for the slower discharge of a battery when a vehicle needs more acceleration at restart. This mode of operation is particularly beneficial in public transportation where frequent start-stops are required but range is not an issue. Aowei Technology here in Shanghai builds city buses with super capacitors and supports the National Engineering Research Center for Super Capacitor Systems for Vehicles. They also use this technology for subway trains. Our team has successfully applied this technology to the streetlights in the Olympic Center in Beijing and shuttles at World Expo in Shanghai. These super capacitors are all built with mesoporous materials.

**Figure 5:**
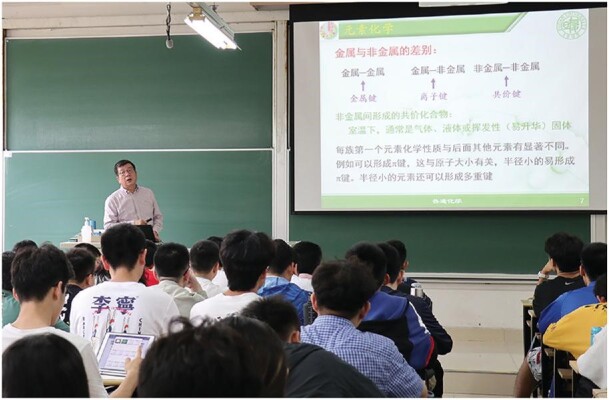
Prof. Zhao teaches general chemistry to undergraduate students (*courtesy: Fudan University*).

## PERSPECTIVES ON RESEARCH AND EDUCATION

***NSR**:* Basic science research has received much attention in recent years, but media and researchers often emphasize application. What are your thoughts on that?

*Zhao:* We often hear news reports of bottlenecks in science or technology. Many bottlenecks exist now due to the lack of innovation in technology. The reason we lack innovation, in my opinion, is that we do not adequately promote the spirit of science. This spirit is centered on always asking why and exploring new knowledge with logic and evidence. As a society, we have intelligent people and resources for research and discovery. What we lack is a culture of asking a question and building systematic knowledge to answer it. Research should be motivated by the new knowledge itself, not any material gains.

I am a chemist so let's use chemistry as an example. Chemistry existed for a long time in alchemy. When people were motivated by gaining gold as wealth, it was not science. Chemistry became science in 1661 when Robert Boyle wrote the first chemistry textbook in which he started investigating matter and change. So in order to advance science, we need to look past material gains and only focus on asking and answering.

Education driven by gaining wealth will not produce the next generation of innovators.

—Dongyuan Zhao

**Figure 6:**
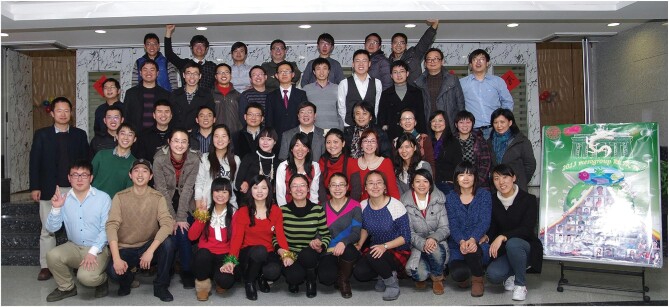
Group photo (*courtesy: Zhao Research Group*).

***NSR**:* That is a great answer. The next question is about education. Along with your research activities, you also give lectures to undergraduate students every year. Why do you think it is important?

*Zhao:* As a university professor, my duty is creating and spreading knowledge. Research in basic science is how new knowledge is created. Teaching is how I fulfill the duty of spreading knowledge.

Every professor can teach the contents of the textbooks, but I think I can also show students the spirit of innovation. I just mentioned how the lack of innovation creates bottlenecks. Education driven by gaining wealth will not produce the next generation of innovators. I want to inspire students to look beyond wealth and see the excitement on scientific frontiers. In this regard, I think I can make more of a contribution because I have been pushing the boundaries with my research. The next generation of scientists must have the spirit of innovation if we want to solve some of these bottleneck problems.

***NSR**:* In recent years, many new fields have appeared in interdisciplinary sciences. Materials science is a good example. When students choose a major related to this for undergraduate or graduate study, they may see three options: physics, chemistry and materials science. What advice can you give them in making this choice?

*Zhao:* The 20th century was the century of quantum mechanics. Now we seem to have a fairly established scientific framework, so the 21st century is the century of fine tuning. Along this path, the classifications of scientific fields are becoming more complicated. The boundaries between many fields are artificially defined. This trend, in my opinion, is counterproductive. In order for science to advance freely, we need to break these boundaries. Most areas of growth are located in the space between different fields. Materials science is inherently an interdisciplinary science between physics and chemistry. It did not exist until a few decades ago and now it has grown into a large and highly active field. Since antiquity, it has been possible to measure the progress of society by the progress of materials. So I would recommend materials science for graduate study if one is attracted to cutting-edge technology. For a high-school student choosing an undergraduate major, I would recommend chemistry.

## Notes

He Zhu (朱禾) is a science and news editor at the editorial office of NSR in Beijing, China.

